# Peripheral sounds elicit stronger activity in contralateral occipital cortex in blind than sighted individuals

**DOI:** 10.1038/s41598-019-48079-3

**Published:** 2019-08-12

**Authors:** Maria Bianca Amadeo, Viola S. Störmer, Claudio Campus, Monica Gori

**Affiliations:** 1 U-VIP: Unit for Visually Impaired People, Istituto Italiano di Tecnologia, Genova, Italy; 2 Department of Informatics, Bioengineering, Robotics and Systems Engineering, Università degli Studi di Genova, Genova, Italy; 3 Department of Psychology and Neuroscience Graduate Program, University of California San Diego, San Diego, USA

**Keywords:** Auditory system, Sensory processing, Visual system

## Abstract

Previous research has shown that peripheral, task-irrelevant sounds elicit activity in contralateral visual cortex of sighted people, as revealed by a sustained positive deflection in the event-related potential (ERP) over the occipital scalp contralateral to the sound’s location. This Auditory-evoked Contralateral Occipital Positivity (ACOP) appears between 200–450 ms after sound onset, and is present even when the task is entirely auditory and no visual stimuli are presented at all. Here, we investigate whether this cross-modal activation of contralateral visual cortex is influenced by visual experience. To this end, ERPs were recorded in 12 sighted and 12 blind subjects during a unimodal auditory task. Participants listened to a stream of sounds and pressed a button every time they heard a central target tone, while ignoring the peripheral noise bursts. It was found that task-irrelevant noise bursts elicited a larger ACOP in blind compared to sighted participants, indicating for the first time that peripheral sounds can enhance neural activity in visual cortex in a spatially lateralized manner even in visually deprived individuals. Overall, these results suggest that the cross-modal activation of contralateral visual cortex triggered by peripheral sounds does not require any visual input to develop, and is rather enhanced by visual deprivation.

## Introduction

Recent findings challenge the assumption that visual cortex is solely processing visual information, as several studies have revealed that nonretinal inputs can trigger neural responses in areas traditionally assumed to be visual in sighted individuals. These activations in occipital cortex have been shown to be elicited by simple auditory stimuli, such as a tone or noise burst e.g.^1^, and also by more complex naturalistic sounds^2^. Other research has demonstrated that peripheral sounds can modulate visual-cortical responses in a spatially selective way. For example, hearing a salient sound on the left side of space elicits a neural response over the right visual cortex and vice versa^3^. In sighted individuals, these lateralized changes over occipital areas have been studied using cross-modal exogenous attention tasks in which peripheral sounds are followed by a visual target either at the same or opposite location as the sound, and have shown that the neural effects are associated with enhanced visual performance at the sound’s location^3,4^. Based on these results, these lateralized enhancements over visual areas have been interpreted as indexing the reflexive orienting of cross-modal spatial attention to the sound’s location. Interestingly, similar lateralized changes over occipital cortex have also been observed in purely auditory tasks – where the observer never sees a visual stimulus^3,5^. This has been taken as evidence that salient sounds can trigger visual-cortical activity reflexively, independently of visual inputs or the task being performed.

What underlies these spatially lateralized audio-visual effects? One possibility is that this cross-modal spatial mapping between audition and vision emerges with experience, i.e., that exposure to co-localized sounds and visual inputs is necessary. Another possibility is that such mapping is inherent to the organization of the occipital cortex, reflecting a built-in mechanism of spatial attention across modalities which does not depend on audio-visual inputs. To test these alternative hypotheses, we here examined congenitally blind and sighted individuals and compared the lateralized response over visual areas triggered by peripheral, salient sounds. Blindness is a natural condition which offers valuable insights into the functional role of cross-modal interactions and their emergence or modulation due to sensory impairment. While there is abundant research showing that blind individuals show strong and reliable responses to sounds in visual cortex^6–11^, it is unknown whether peripheral, task-irrelevant sounds would elicit spatially lateralized responses in visual areas of blind individuals. This is important because it would suggest that spatial information – at least at the level of the hemifield (left vs. right) – is coded in the visual cortex, regardless of input modality and regardless of visual or cross-modal experience.

Previous research in blind people shows mixed results with regards to spatial processing. On the one hand, research in blind individuals has shown that the brain is highly plastic and can adopt compensatory mechanisms following visual deprivation. The visual cortex becomes, to some extent, colonized by the auditory and somatosensory systems^12,13^, and the enhanced performance of blind people in some behavioral tasks seems to be related to the recruitment of occipital areas deprived of normal visual inputs^14,15^. Indeed, congenitally blind subjects have enhanced skills in auditory pitch discrimination^16^, localization of peripheral sounds in the horizontal plane^17–19^, and they are able to form auditory spatial topographical maps^20,21^. On the other hand, a growing body of literature raises some doubts about the extent of cross-modal plasticity in the case of sensory loss. Disordered auditory spatial maps have been reported in the superior colliculus of owls reared with distorting visual prisms^22^, and in young ferrets totally deprived^23^. Turning attention to humans, comparable but transitory effects have also been demonstrated^24,25^, suggesting that modified visual inputs can affect auditory spatial processing. Together, the literature points to complex cross-modal interactions in blind individuals that may depend on stimulus type and the exact task used. To date, however, it is unclear whether peripheral sounds activate contralateral occipital cortex in the blind, as has been observed in sighted individuals.

Here, we examine whether and to what extent cross-modal activation of the contralateral occipital cortex is present in blind individuals and thus mediated by visual experience. We focus on an ERP component termed the Auditory-evoked Contralateral Occipital Positivity (ACOP) that appears between ~200–450 ms after the onset of a peripheral sound^3^. If the sound-induced lateral effects over occipital cortex emerge independently of visual experience, we would predict they also occur in blind individuals. As the ACOP has been found robustly and mostly independently of task in sighted individuals^3^, it seems plausible that these spatially lateralized responses also occur in blind individuals. In particular, if this lateralized enhancement represents the neural signature of the reflexive orienting of spatial attention – regardless of input modality – one might assume that this component is elicited in blind individuals as well, consistent with research showing enhanced spatial attention abilities in blind people^11,17,19^. Alternatively, if this cross-modal activation depends on an observer’s experiences with spatially overlapping audio-visual inputs, we would predict that it is absent in blind individuals. To test these hypotheses, ERPs were recorded in 12 sighted and 12 blind subjects during a unimodal auditory task as in previous studies investigating the ACOP^3^. Participants listened to a stream of sounds presented in random order and at unpredictable times, and were instructed to press a button every time they heard a central target tone, while ignoring peripheral noise bursts. Results show that peripheral noise bursts elicit a stronger ACOP in blind compared to sighted participants, indicating that peripheral sounds enhance neural activity in visual cortex in a spatially lateralized manner even when visual input is absent during development.

## Methods

### Participants

A group of 12 congenitally blind participants (mean age: 37 ± 15 yo; F = 7) and 12 age and gender-matched sighted participants (31 ± 8 yo; F = 7; t-test comparing age between groups: t_18.2_ = 1.18, p = 0.3) took part in the study (see Table 1 for details). All participants reported no history of neurological or cognitive deficits. The research protocol was approved by the ethics committee of the local health service (Comitato Etico, ASL3 Genovese, Genova, Italy) and conducted in line with the Declaration of Helsinki. Written informed consent was obtained prior to testing.

**Table 1:** Clinical details of the blind group (N = 12).

Participant	Age	Gender	Pathology	Blindness onset	Residual vision
S1	52	M	Retinopathy of Prematurity	Birth	Light and shadow
S2	77	F	Retinitis Pigmentosa	Birth	No vision
S3	62	F	Atrophy of the eyeball	Birth	Light and shadow
S4	25	M	Leber amaurosis	Birth	No vision
S5	52	F	Retinitis Pigmentosa	Birth	No vision
S6	58	M	Uveitis	Birth	No vision
S7	59	M	Glaucoma	Birth	Light and shadow
S8	42	F	Glaucoma	Birth	Light and shadow
S9	28	F	Retinopathy of Prematurity	Birth	No vision
S10	27	F	Retinopathy of Prematurity	Birth	No vision
S11	24	F	Glaucoma	Birth	No vision
S12	27	F	Microphthalmia	Birth	No vision

The table shows chronological age, gender, pathology, age of blindness onset, and residual vision at testing for each participant.

### Stimuli and procedure

**Figure 1:**
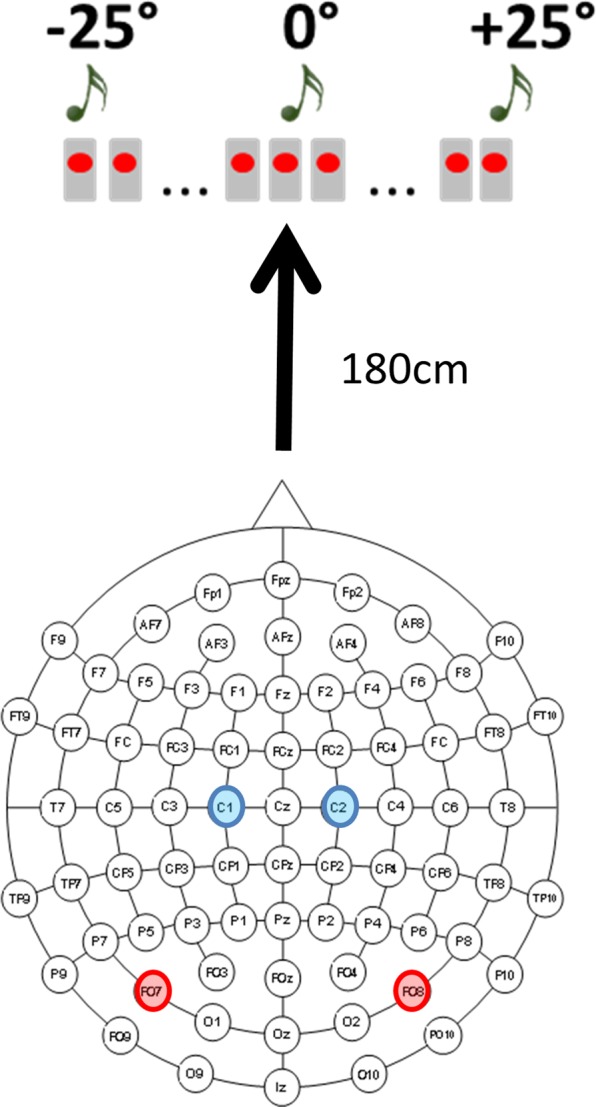
Experimental Setup and electrode montage. Participants were blindfolded and sat in a silent room, 180 cm away from the center of an array of 23 speakers spanning ±25° of visual angle (with 0° representing the central speaker, negative values on the left, and positive values on the right). In the analysis, left (PO7) and right (PO8) parieto-occipital electrodes and left (C1) and right (C2) central electrodes were considered.

Participants were blindfolded and sat in a silent room, 180 cm away from the center of an array of 23 speakers spanning ±25° of visual angle (with 0° representing the central speaker, negative values on the left, and positive values on the right; Fig. 1).

They listened to a stream of sounds that were presented in random order and at unpredictable times (i.e. variable inter-stimulus-interval: 2000–2500 ms). The auditory stream included task-irrelevant bursts of pink noise (83 ms duration, 0.5–15 kHz, 60 dB SPL) delivered from the left or right sides (i.e. ± 25° eccentricity), and 1000 Hz target tones (83 ms duration, 60 dB SPL) delivered from the center i.e. 0° eccentricity; similar to^3^, Exp. 4. Participants were instructed to press a button every time they heard a central target tone, while ignoring the peripheral noise bursts. The experiment consisted of 5 blocks of 128 trials. In each block, the proportions of noise bursts and tones were set to 55% and 45% respectively. We measured reaction times (RT), as the time between target tone and button press (button press was allowed only after central target tones and it was required to proceed with the task, i.e. no false positives or omissions could be recorded). Before testing, all subjects were directed to maintain a stable head position and to fixate straight ahead. However, head and body orientation was continuously monitored during the experiment by the researchers (no differences emerged between groups).

### EEG data acquisition and pre-processing

High-density EEG was recorded from 64 scalp electrodes using the Biosemi ActiveTwo EEG System (Fig. 1). Preamplifiers in each electrode were used to reduce noise between the electrode and the amplification/digitization system (BioSemi ActiveTwo, BioSemi B.V. Amsterdam), allowing high electrode impedances. Electrode offsets were kept below 35 mV. The continuous EEG was recorded referenced to a Common Mode Sense (CMS) active electrode and a Driven Right Leg (DRL) passive electrode, which replace the ground electrodes used in conventional systems. CMS and DRL form a feedback loop, thus rendering them references. A first-order analog anti-aliasing filter with a half-power cutoff at 3.6 kHz was applied (see www.biosemi.com). Data were sampled at 512 Hz (2048 Hz with a decimation factor of 1/4) with pass-band from DC to 134 Hz. In order to monitor horizontal eye movements, two additional electrodes were placed at the left and right outer canthi for EOG recording and trials showing horizontal ocular movements were discarded by visual inspection. EEG was filtered between 0.1 and 45 Hz and filtered data were referenced to the average of left and right mastoids.

### ERPs and statistical analysis

The ERP analyses followed closely the procedures employed in a prior study investigating the ACOP component in sighted individuals^3^. Thus, the EEG analysis focused on the ERPs triggered by the task-irrelevant noise bursts. For each subject, a minimum of 166 stimuli per position (left and right) after artifact rejection was required. On average, there were 349 trials per subject across left- and right sound trials. ERPs elicited by the left and right noise bursts were collapsed across sound position (left, right) and hemisphere of recording (left, right) to obtain ERP waveforms recorded on the hemisphere contralateral and on the hemisphere ipsilateral with respect to stimulus location. Lateralized ERP waveforms were calculated as the relative difference between the contralateral and ipsilateral responses. Based on previous literature^3,26^, we focused on two posterior electrode sites (PO7/PO8) for the ACOP analysis, and on two central electrode sites (C1/C2; Fig. 1B) to examine auditory processing. Mean ERP amplitudes at parietal-occipital electrode sides (PO7/PO8) were computed by averaging the voltage in a 250–500 ms time window after the onset of the peripheral sound. For each group, scalp topographies of mean ERP amplitude in the 250–500 ms time window were created separately for the left and right sounds (−25° and +25°), before averaging the two hemifield responses. The window was chosen based on previous literature^3^ in order to investigate the ACOP component in blind compared to sighted people.

To examine whether the ACOP was present in each group, the resulting mean amplitudes in the 250–500 ms time window were analyzed in an omnibus ANOVA with group (sighted vs blind) as a between-subjects factor, and electrode site (contralateral vs ipsilateral relative to the sound location) as a within-subjects factor. Planned pairwise comparisons were conducted with two-tailed t-tests to see whether the ACOP was reliably present in each group. As the ACOP is defined by the relative difference between the contralateral and ipsilateral activation, to investigate the difference between groups we also ran a two-tailed t-test on the lateralized mean amplitude difference at parietal-occipital electrode sides in the selected time window. In order to address any latency group differences, we computed the average onset, offset, and duration for contralateral and for ipsilateral electrodes and compared them between sighted and blind individuals. Specifically, for each subject, we independently considered the average ERP of contralateral and ipsilateral electrodes. We computed the mean and the SD of the ERP during the baseline. Then, for each time point within the 250–500 ms time window, we performed a Z-test to compare the ERP at that time with the baseline activity, applying FDR correction to p-values. We retained as onset of the component the first time within the 250–500 ms corresponding to a significant deflection from the baseline (p < 0.05 after FDR correction). Similarly, the offset was estimated as the latest time within the time window different from baseline. The duration was given by the difference between the offset and the onset of the deflection. Next, we compared the latency of onset and offset, considering the responses ipsilateral and contralateral relative to the sound location separately. Similarly, we also compared across groups the duration of the ipsilateral and contralateral waveforms. Furthermore, since the blind right and left visual cortices have shown different roles (e.g. with relation to language and memory processing in the left visual cortex), we checked for hemispheric effects in the blind group by analyzing ERP elicited at parietal-occipital electrode sides (PO7/PO8) by the left (−25°) and right sounds (+25°) separately. Thus, we performed paired t-tests to compare the ERP mean amplitude in the ACOP time window between PO7 and PO8 when they were ipsilateral relative to the stimulus position in space, and between PO7 and PO8 when they were contralateral relative to the stimulus position in space. We similarly compared the ACOP (contralateral-ipsilateral) elicited when the stimulus was delivered from the left, with the ACOP (contralateral-ipsilateral) elicited when the stimulus was delivered from the right. For the blind group, we also investigated whether the ACOP amplitude was influenced by blindness duration through correlational analyses (since they were congenitally blind subjects blindness duration coincided with chronological age).

Moreover, a two-tailed t-test with group (sighted vs blind) as a between-subjects variable was conducted to compare reaction times between sighted and blind people.

To exclude the presence of confounding effects due to eye-movement, we also performed t-tests to test whether the mean response of the eye deviation measured by EOG significantly differs from zero within each group, and whether it differs across groups. The irrelevance of eye-movement is evident in plots showing the amplitude of ocular movements calculated as the difference between the left and the right EOG for blind and sighted subjects.

## Results

**Figure 2:**
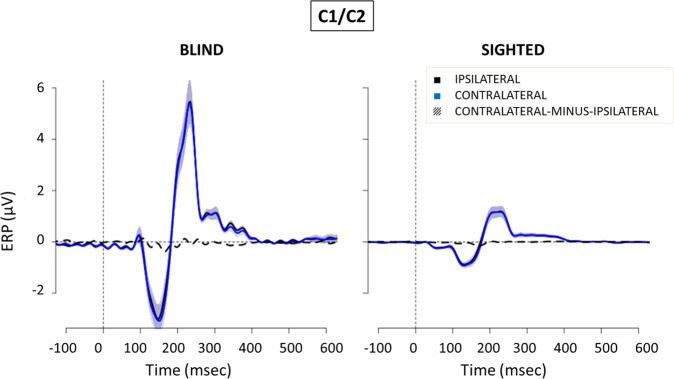
ERPs (mean ± SEM) elicited by peripheral noise bursts at central (C1/C2) electrodes in blind (left) and sighted (right) subjects. In blue, ERPs collapsed over central scalp sites contralateral to the side of the stimulus presentation. In black, ERPs collapsed over central scalp sites ipsilateral to the side of the stimulus presentation. Dashed line, contralateral minus ipsilateral difference amplitude. On the x-axis, 0 is sound onset.

**Figure 3:**
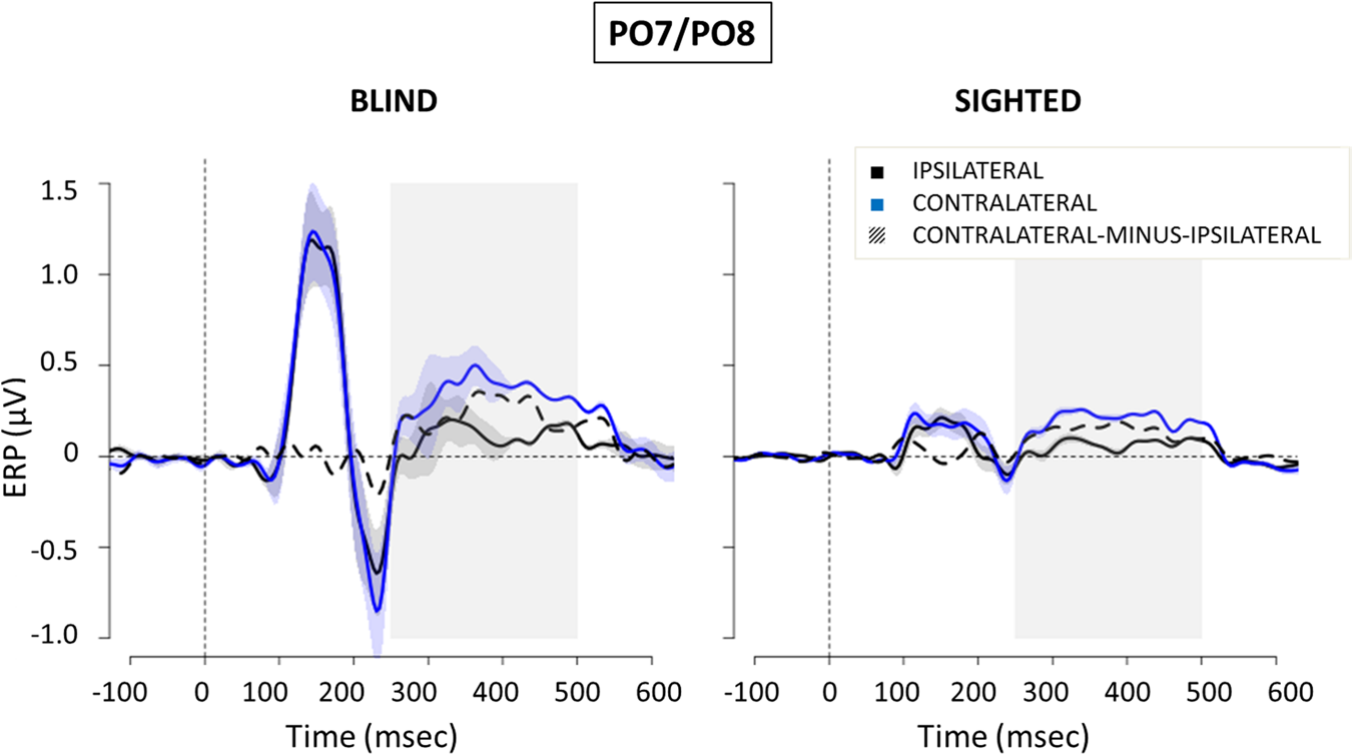
ERPs (mean ± SEM) elicited by peripheral noise bursts at parieto-occipital (PO7/PO8) electrodes in blind (left) and sighted (right) subjects. In blue, ERPs collapsed over parieto-occipital scalp sites contralateral to the side of the stimulus presentation. In black, ERPs collapsed over parieto-occipital scalp sites ipsilateral to the side of the stimulus presentation. Dashed line, contralateral minus ipsilateral difference amplitude. The gray area indicates the time window of the ACOP (250–500 ms). On the x-axis, 0 is sound onset.

The EEG analysis focused on the ERPs triggered by the task-irrelevant noise bursts in order to investigate the ACOP component in blind and sighted participants. ERPs elicited by noise bursts at central (C1/C2) and parieto-occipital (PO7/PO8) electrodes of blind and sighted subjects are reported in Figs 2 and 3 respectively.

Several typical auditory ERP components were observed in the initial 200 ms following cue onset in central area (Fig. 2), including the N1 (110–140 ms) and a slightly later P2 (210–250 ms) over bilateral scalp regions. These negative ERP components reflect modality-specific sensory processing within the auditory cortex^26^ and, as expected e.g.^27^, are enhanced in blind compared to sighted individuals. With regards to the posterior scalp regions (see Fig. 3), the earlier components are still more pronounced in blind than in sighted individuals, in line with previous literature reporting a posterior shift in the scalp topography of the auditory ERP responses following blindness^18,28,29^. For both groups, in the initial 200 ms following sound onset no differences emerged between the ERP waveforms recorded over the posterior sites contralateral and ipsilateral to the auditory cue. However, for both sighted and blind participants a stronger activation in contralateral compared to ipsilateral scalp sites appeared between 250 and 500 ms, as evident from the timing and amplitude of difference waveform created by subtracting the ERP recorded ipsilaterally from those recorded contralaterally (Fig. 3, dashed line).

**Figure 4:**
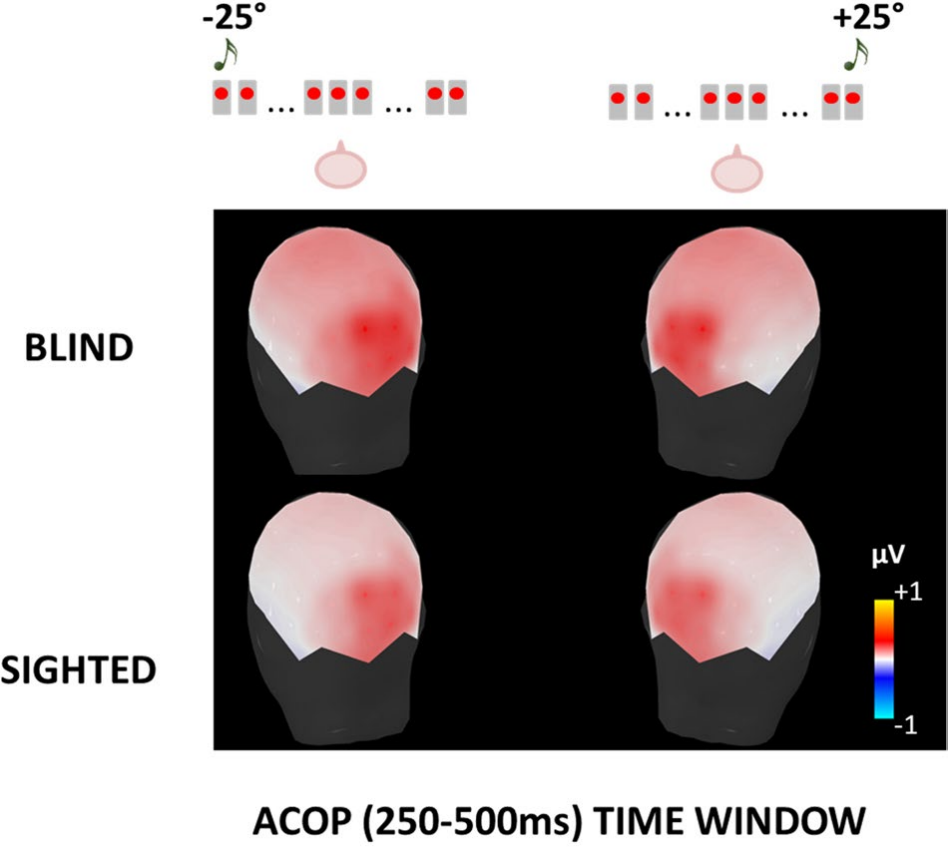
Scalp maps of the mean ERP amplitude in the selected time window (250–500 ms) after peripheral noise bursts, obtained before averaging the two hemifield responses. The stimulus was presented in space from either − 25° (i.e. left side of the subject; see left panel) or +25° (i.e. right side of the subject; see right panel). First row represents blind subjects, second row represents sighted subjects.

In Fig. 4, we show the scalp maps of the mean ERP amplitude in 250–500 ms time window for blind (Fig. 4 top) and sighted (Fig. 4 bottom) subjects, when noise bursts were presented from either the left (−25°; Fig. 4 left) or right (+25°; Fig. 4 right). The topographies of the mean ERP amplitude in the ACOP time window are shown before averaging the two hemifield responses.

**Figure 5:**
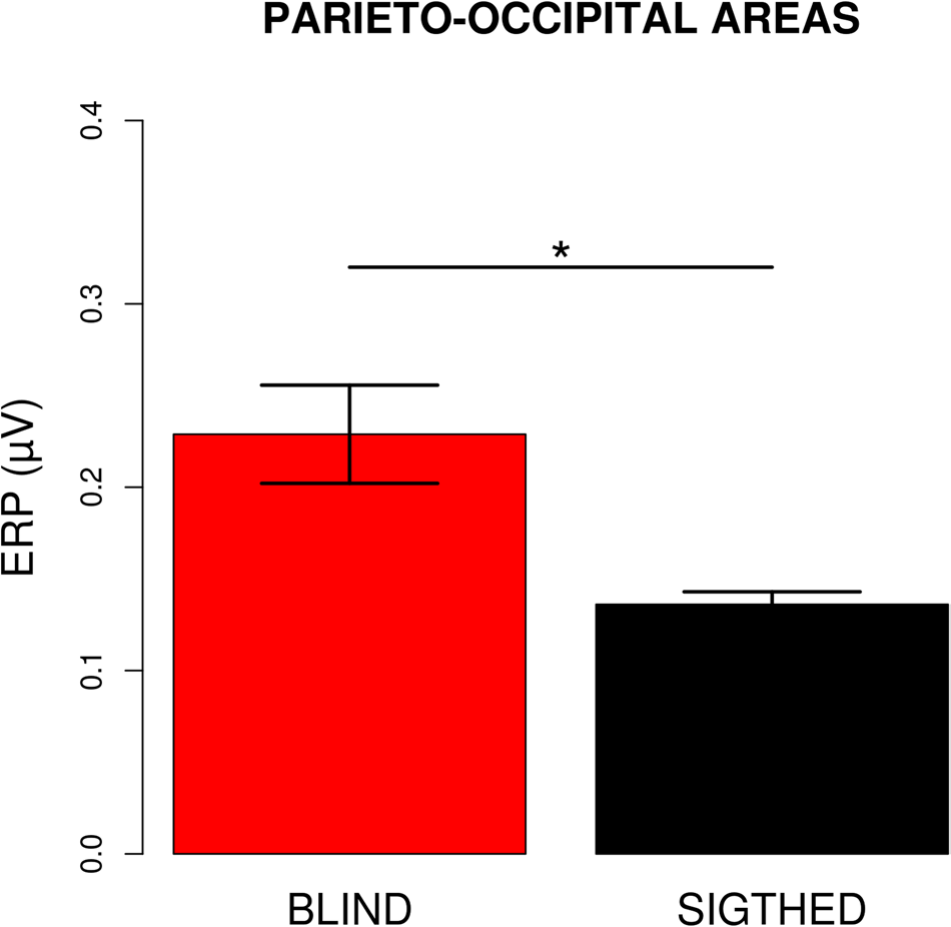
Lateralized (contralateral-minus-ipsilateral) ERP amplitude (mean ± SEM) for blind (left) and sighted (right) group in the time window between 250–500 ms after peripheral noise bursts. The star indicates a significant difference between the groups (p < 0.05.)

Statistical analysis to investigate whether the ACOP was present in each group revealed a significant interaction (F_1,22_ = 11.25, p = 0.002, Generalized Eta Squared - GES = 0.01) between group (sighted vs blind) and electrode site (contralateral vs ipsilateral relative to the sound location). Pairwise comparisons revealed a greater positivity over the contralateral relative to the ipsilateral posterior-occipital scalp in both sighted (t_11_ = 19.85, p < 0.001, d = 3.02) and blind (t_11_ = 8.54, p < 0.001, d = 0.82) groups. Thus, similar to sighted individuals, blind participants showed the presence of an ACOP, as noise bursts elicited a significant positive activation over contralateral relative to ipsilateral scalp sites with respect to the stimulus position in space. A planned pairwise comparison of the contralateral-minus-ipsilateral waveform in the same time window and sites revealed a larger amplitude difference in blind compared to the sighted (t_12.4_ = 3.35, p = 0.005, d = 1.37; see Fig. 5), suggesting that the ACOP was more pronounced in visually impaired people. Although the GES of the omnibus ANOVA indicates a mild effect size, the Cohen’s d for t-tests reveal a larger effect size. We checked whether the ACOP was stronger over one hemisphere, but did not observe differences between left and right electrode sites. In particular, the right and left hemisphere processed the noise bursts similarly from the left (i.e. −25°) and the right (i.e. +25°) side (t-test to compare PO7 with PO8 when they were ipsilateral relative to the stimulus position in space: t_11_ = 1, p = 0.3; t-test to compare PO7 with PO8 when they were contralateral relative to the stimulus position in space: t_11_ = 0.02, p = 0.98). Moreover, the ACOP elicited when the stimulus was delivered from the left was similar to the ACOP elicited when the stimulus was delivered from the right (t_11_ = 0.05, p = 0.6).

**Figure 6:**
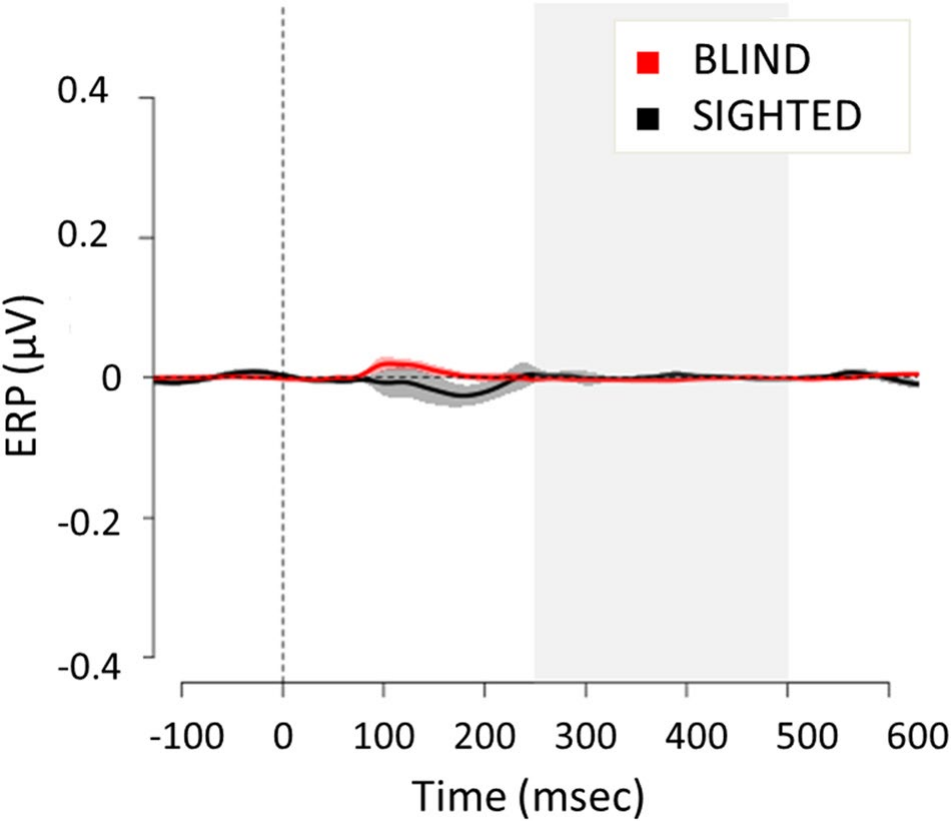
Mean ( ± SEM) amplitude of ocular movements calculated as the difference between the left and the right EOG for blind (red) and sighted (black) subjects. On the left, average of trials in which the stimulus was delivered from −25° (i.e. from left); on the right, average of trials in which the stimulus was delivered from +25° (i.e. from right). On the x-axis, t = 0 is sound onset. The shaded area delimits the selected time window (250–500 ms).

Moreover, we did not find significant latency differences between groups, neither for the onset nor for the offset of the contralateral (onset: t_22_ = 0.79, p = 0.4; offset: t_22_ = −1.32, p = 0.2) and ipsilateral (onset: t_22_ = 0.65, p = 0.5; offset: t_22_ = −0.16, p = 0.9) waveforms within the ACOP time window. Also the duration of the ipsilateral and contralateral component within the selected time window did not differ across groups (for contralateral t_22_ = −1.17, p = 0.3; for ipsilateral: t_22_ = −0.67, p = 0.5). We can exclude that the effects originated from spurious eye-movement towards the apparent location of the stimulus (see Fig. 6). Indeed, the average response of the eye deviation measured by EOG is very low and did not significantly differ from zero neither for sighted (t_11_ = 1.63, p = 0.1) nor for blind (t_11_ = 1.04, p = 0.3) participants. Moreover, the average ocular deflection recorded by EOG is similar between blind and sighted (t_12.4_ = 1.41, p = 0.2). Finally, no differences were observed for RTs comparing blind (mean RT = 344.6 ± 92.8 ms) and sighted (mean RT = 348.2 ± 53.1 ms) groups (t_17.5_ = −0.12, p = 0.9). As regards the impact of blindness duration on the ACOP amplitude, there was no significant association between the two variables (r = −0.04, p = 0.9).

In Supplementary Materials, we show the ERPs elicited by central targets at central (C1/C2; Supplementary Figure 1) and parieto-occipital (PO7/PO8; Supplementary Figure 2) electrodes for blind and sighted subjects. As expected for the processing of central sounds (e.g.^18,27^),there are no evident differences between the two groups at central scalp sites. However, the amplitude of the N1 elicited by central tones is higher than the amplitude of the same component elicited by unpredictable task-irrelevant noise bursts. This is also in line with other research^18^, showing that the N1 amplitude progressively decreases in response to sounds increasingly distant from the attended speaker.

## Discussion

The present study investigated whether the lateralized enhancement of visual cortex by peripheral sounds previously observed in sighted individuals is also present in the congenitally blind. If the sound-induced changes in visual cortex activity depend on a lifetime experience of spatially aligned sounds and sights, we would expect that no such cross-modal activation occurs in blind individuals. However, if these lateralized changes in visual activity are an amodal signature of spatial representation/orienting independently of audio-visual experiences, we would expect these effects to be present in blind individuals as well. Our results support the latter hypothesis, showing that the lateralized activation of visual cortex (i.e., ACOP) was evident in blind people and even more pronounced compared to the same response in sighted individuals. These results provide two important points of discussion. The first implication involves the cross-sensory interactions between vision and audition at cortical sites: the late auditorily-induced activation of contralateral visual cortex does not require visual input to develop. The second implication sheds light on the structural and functional organization of the visual cortex in blind people: this is the first time that a response selective to the spatial position of a sound is reported over visual cortex of blind individuals.

The ACOP has been previously studied in the context of the reflexive orienting of spatial attention in sighted individuals. It has been shown to be elicited by salient peripheral sounds even when the tasks are purely auditory, and when the sounds are both spatially and temporally unpredictable of subsequent relevant target events^3,5^, although it appears to be somewhat sensitive to the spatial predictability of the sounds^30^. The neural generators of the ACOP have been ascribed to the ventrolateral extrastriate visual cortex (Brodmann’s Area BA19, see also results from a ECoG study by^31^). Here, we report a more pronounced ACOP over visual cortex of blind compared to sighted individuals. The stronger visual response to auditory stimuli in blind is in agreement with other studies showing that compensatory mechanisms are triggered by visual deprivation, driving brain structures normally involved in the processing of visual information to be activated during tactile^12,32–34^, auditory^9,13,18^, memory^35,36^ and language-related^37,38^ processing in congenitally blind participants. Specifically, the recruitment of ventral extrastriate occipital areas has been reported in congenitally blind people during auditory localization tasks (e.g.^13,14,39^), supporting the implication of these visual regions in spatial hearing following blindness. Actually, visual areas are not only found to be activated during auditory tasks but the magnitude of activation is also associated with localization abilities of blind subjects^14,40,41^. For example, early blind people localize sounds more accurately than sighted controls under monaural conditions^17^, and their activation in right-hemisphere striate and ventral extrastriate areas correlates with the performance in a pointing task to monaurally presented sounds^14^. In line with that study, we found stronger activation over occipital scalp sites of blind compared to sighted people in response to peripheral sounds. Different from previous work, we observed a response under binaural conditions that was selective for the spatial position of the sound in terms of hemifield (left vs. right). The divergence from prior work is likely due to differences in task (i.e. the stimuli in our experiment were task-irrelevant), measurement (fMRI vs EEG), or to other differences between the two occipital activations investigated. In our experiment we investigated an involuntary response which has previously been shown to activate contralateral occipital cortex in sighted individuals. Thus, in both studies, the higher activation in blind compared to sighted individuals may be explained by similar mechanisms of cross-modal plasticity that strengthen some multisensory neural connections which are present in sighted individuals as well. If we performed our experiment under monaural condition, we may expect that blind people activate the contralateral occipital sites even more in the monaural relative to the binaural condition.

Previous research also showed better auditory attention abilities in blind people^18,42^, and a superior ability of blind people to localize sounds particularly when those occur in the periphery^17,18,43^. For example, Röder *et al*.^18^ and Fieger *et al*.^43^ investigated auditory spatial tuning in early and late blind individuals. They asked participants to detect infrequent sounds at an attended location, which was either in the center or in the periphery, and found that early auditory processing was enhanced more so for peripheral locations than central ones. While this study investigated group differences in *voluntary* auditory attention (i.e., participants were asked to attend to particular sound locations in the visual field), challenging a direct comparison with the current study in which participants were asked to ignore the peripheral sounds, there nonetheless appear to be interesting parallels to the current findings. For example, this may suggest that any differences in sound processing between blind and sighted individuals are most strongly pronounced at peripheral visual field locations, both for voluntary and involuntary attention. Moreover, according to Röder *et al*.^18^, in early blind individuals the better performance in the periphery was sustained by a more sharply tuned N1 component, characterized by a more posterior distribution. Similarly, although we did not measure behavioral performance related to the peripheral noise bursts, the auditory N1 elicited by the peripheral task-irrelevant stimuli in our study was more pronounced and posterior in congenitally blind than sighted individuals (see Fig. 2 for central electrodes and Fig. 3 for posterior electrodes). In addition, the lack of group differences in performance for the attended central stimuli in both Roder *et al*.^18^ and Fieger *et al*.^43^ is in line with similar reaction times to the central target tones between sighted and blind people involved in our study.

All previous research on the ACOP looked at sighted individuals only and found that a larger ACOP is associated with better visual discrimination accuracy at the sound’s location (e.g.,^3,4^). One interesting possibility that arises from the present results is that the ACOP is not only associated with enhanced visual processing at the sound’s location, but also with enhanced auditory spatial processing. Since several studies have reported that enhanced spatial hearing abilities of blind individuals are subserved by crossmodal plasticity (see also^44^), we may speculate that an increased ACOP amplitude following visual deprivation may reflect enhanced abilities of congenitally blind individuals to reflexively orient spatial attention to the sound’s location. If this is the case, this would help blind individuals in information processing in multisensory environments.

One recent study that tested complex spatial representations using the space bisection task^45^ found a weaker and non-lateralized response to sounds in the occipital areas of blind compared to sighted individuals. While this result may appear conflicting to the results reported here, we think they can be well explained by differences between the tasks used. The previous study used a difficult space bisection task that requires spatial representations in Euclidian coordinates, strong spatial skills in terms of memory and attention, and taxes a sophisticated, and well-calibrated spatial auditory map. In contrast, the task used here does not stress the construction of complex spatial metrics at all. Thus, the present results are overall in agreement with these previous studies highlighting some limits of neuroplasticity.

Several anatomical routes that may mediate auditory responses in occipital cortex have been identified, including direct pathways between lower-level unimodal regions^46,47^ and indirect feedback connections from higher multisensory regions to unimodal sensory regions^48,49^. Given the late onset of the ACOP signal (i.e. 250 ms after the auditory cues) and the relatively late stage in the visual processing hierarchy that it has been localized to, it seems unlikely that the ACOP is mediated by direct pathways between the auditory and the visual cortices. Rather, the data are consistent with the involvement of longer hierarchical pathways and higher-level multisensory regions prior to activating the visual cortex. Although we cannot infer what the exact cortical structures are underlying the ACOP, the fact that we observe an ACOP also in blind people suggests that the specific pathways involved in eliciting the ACOP do not require the visual input to develop but are instead enhanced due to sensory impairment. Thus, the present findings add evidence to the hypothesis of mutual interaction between *supramodal* organization and *cross-modal* plasticity of the brain, that can be considered as the “yin and yang” of brain development according to Ricciardi and colleagues (see^50^). Specifically, the occipital activation to sounds previously observed in sighted individuals and found here in blind individuals support the idea that several visual brain regions can develop despite the absence of any visual experience, and can respond to perceptual information independently of the sensory modality that conveys the input (i.e. *supramodal* organization). At the same time, the stronger response in blind compared to sighted people suggests that the lack of visual experience can drive cross-modal reorganization within brain areas that are deprived of their normal visual inputs, and can start responding more strongly to non-visual stimuli (i.e. *cross-modal* plasticity).

One other main insight of the present study involves the spatial lateralization of the auditory ERP component in the visual cortex of blind people. Although the involvement of occipital cortical areas in purely auditory tasks is commonly reported in blind individuals^6–11,51^, previous studies in blindness failed to show neural activity in visual cortex selective to the spatial position of sounds. One study^52^ reported a contralateral activation of the calcarine cortex in one blind echolocator but in response to sounds that contained both clicks and the returning echoes, with respect to control sounds that did not contain the echoes. The spatially lateralized pattern of the occipital activation we observe in blind people enriches the recent body of literature reporting that some retinotopic organization principles are preserved in blind people e.g.^53–55^. Indeed, previous studies based their conclusions on resting state or anatomical connectivity analyses, without actually showing a contralateral activation of the visual cortex of blind people in response to auditory stimulation. Recently, Sourav *et al*.^56^ demonstrated with EEG that one basic feature of the retinotopic organization (i.e. upper versus lower visual field organization), is present in people who were born with total bilateral cataracts and subsequently underwent cataract-removal surgeries. With our experiment, we assert that another fundamental aspect of retinotopic organization (i.e. laterality of visual field) is retained in the visual cortex without the need of visual input. Thus, although visual deprivation can impair the ability of the brain to acquire some skills which require visual calibration (e.g. spatial bisection abilities), our data strengthen the notion that some basic features of retinotopic processing are independent of sensory experience, further supporting the account of *supramodal* cortical organization^50^. In particular, it appears that peripheral sounds can enhance visual-cortical processing in a hemisphere-specific way in blind individuals.

In conclusion, we show that the cross-modal activation of contralateral visual cortex previously observed in sighted individuals does not require any visual experience to develop, but is instead enhanced by visual deprivation. These results indicate multisensory interactions between the visual and auditory cortices do not necessarily depend on a lifelong multisensory experience. Furthermore, the fact that the sound-induced activations over visual areas are spatially lateralized suggests that the visual cortex is inherently organized retinotopically – at least at the level of the hemifield.

## Supplementary information

Supplementary Materials

## Data Availability

The datasets generated during the current study are available from the corresponding author on reasonable request.
