# Supplementary material for: Peripheral sounds elicit stronger activity in contralateral occipital cortex in blind than sighted individuals
Source: Sci Rep. 2019 Aug 12;9:11637. doi: 10.1038/s41598-019-48079-3 (PMC6690873; doi:10.1038/s41598-019-48079-3)
Supplement: Supplementary file 1 — Supplementary Materials [file 41598_2019_48079_MOESM1_ESM.pdf]

Supplementary information

Peripheral sounds elicit stronger activity in contralateral occipital cortex in blind than sighted individuals

Amadeo Maria Bianca[1,2]* Störmer S. Viola[3], Campus Claudio[1], Gori Monica[1]

[1]U-VIP: Unit for Visually Impaired People, Istituto Italiano di Tecnologia, Genova, Italy

[2] Department of Informatics, Bioengineering, Robotics and Systems Engineering, Università degli Studi di Genova, Italy

[3] Department of Psychology, University of California San Diego

* Corresponding author: mariabianca.amadeo@iit.it, Fondazione Istituto Italiano di Tecnologia, Via E. Melen, 83 - 16152 Genova (Italy)

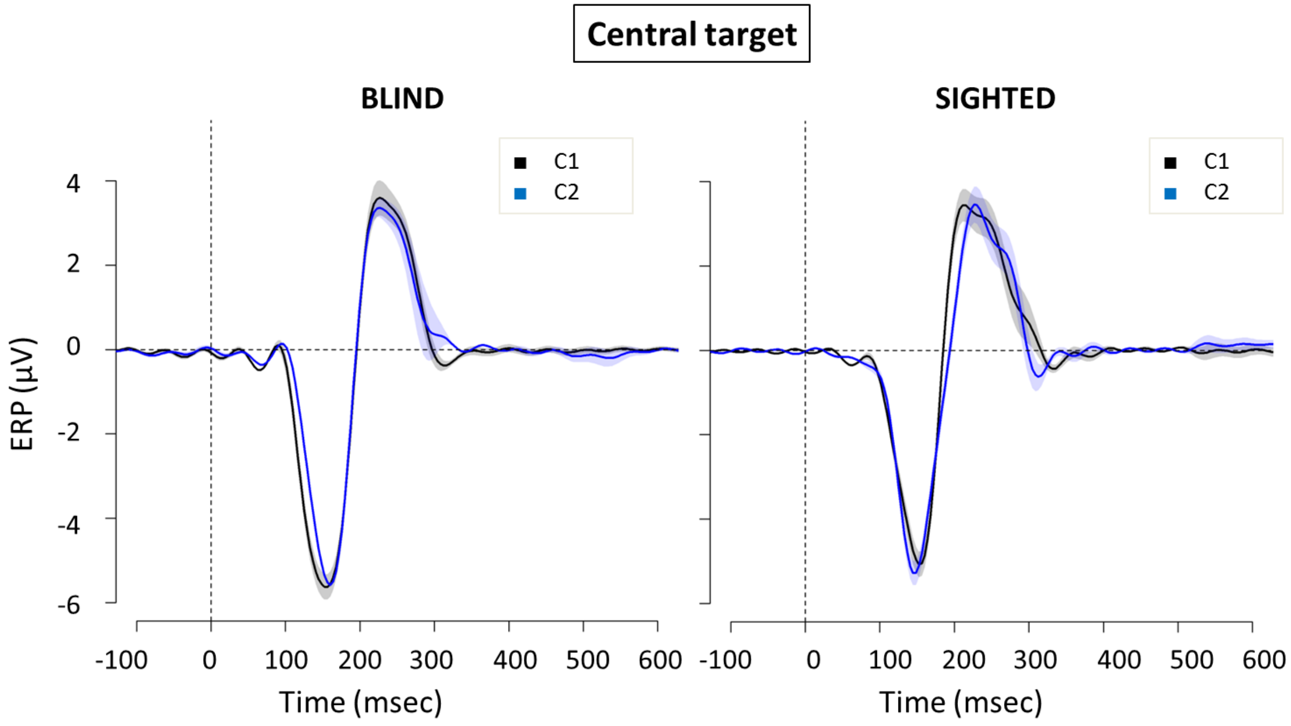

SUPPLEMENTARY FIGURE 1

ERPs (mean ± SEM) elicited by central target tones at central (C1/C2) electrodes in blind (left) and sighted (right) subjects. Black and blue waveforms represent ERPs elicited in C1 and C2 respectively. On the x-axis, t = 0 is sound onset.

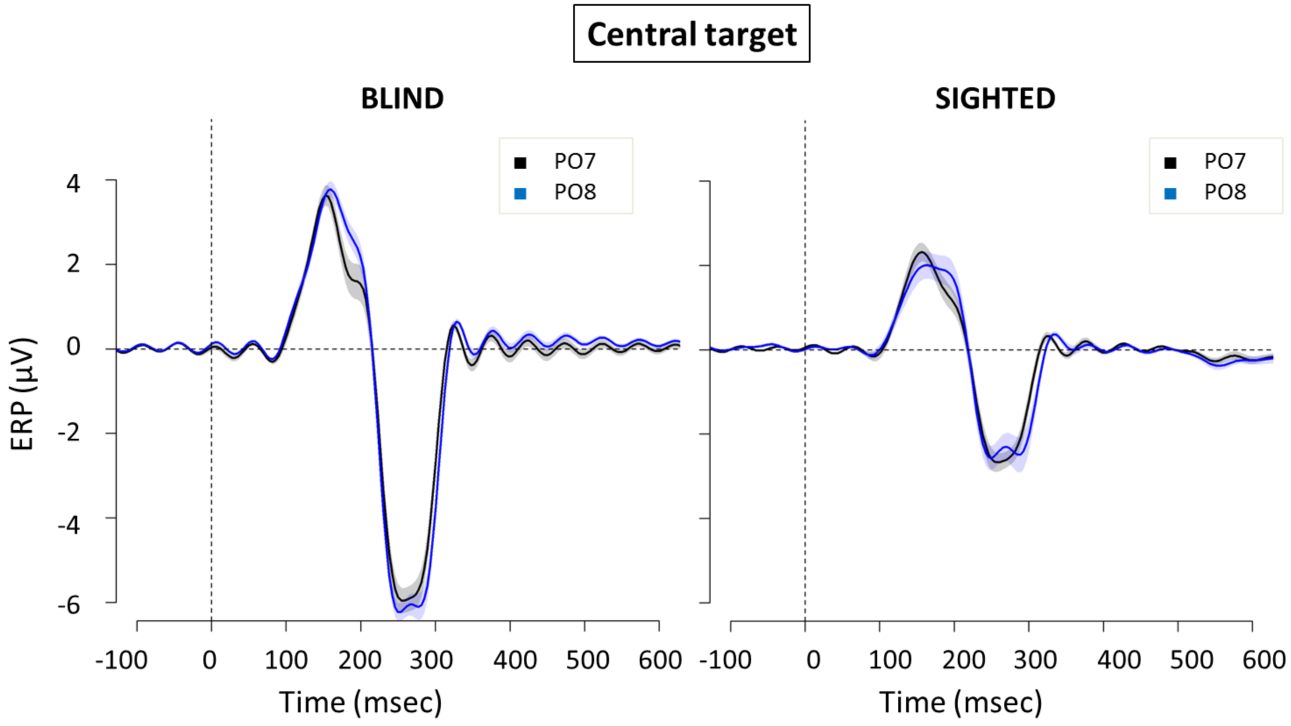

SUPPLEMENTARY FIGURE 2

ERPs (mean ± SEM) elicited by central target tones at parieto-occipital (PO7/PO8) electrodes in blind (left) and sighted (right) subjects. Black and blue waveforms represent ERPs elicited in PO7 and PO8 respectively. On the x-axis, t = 0 is sound onset.
